# A mathematical model for the role of dopamine-D2 self-regulation in the production of ultradian rhythms

**DOI:** 10.1371/journal.pcbi.1012082

**Published:** 2024-05-03

**Authors:** An Qi Zhang, Martin R. Ralph, Adam R. Stinchcombe

**Affiliations:** 1 Department of Psychology, University of Toronto, Toronto, Ontario, Canada; 2 Department of Mathematics, University of Toronto, Toronto, Ontario, Canada; Pázmány Péter Catholic University: Pazmany Peter Katolikus Egyetem, HUNGARY

## Abstract

Many self-motivated and goal-directed behaviours display highly flexible, approximately 4 hour ultradian (shorter than a day) oscillations. Despite lacking direct correspondence to physical cycles in the environment, these ultradian rhythms may be involved in optimizing functional interactions with the environment and reflect intrinsic neural dynamics. Current evidence supports a role of mesostriatal dopamine (DA) in the expression and propagation of ultradian rhythmicity, however, the biochemical processes underpinning these oscillations remain to be identified. Here, we use a mathematical model to investigate D2 autoreceptor-dependent DA self-regulation as the source of ultradian behavioural rhythms. DA concentration at the midbrain-striatal synapses is governed through a dual-negative feedback-loop structure, which naturally gives rise to rhythmicity. This model shows the propensity of striatal DA to produce an ultradian oscillation characterized by a flexible period that is highly sensitive to parameter variations. Circadian (approximately 24 hour) regulation consolidates the ultradian oscillations and alters their response to the phase-dependent, rapid-resetting effect of a transient excitatory stimulus. Within a circadian framework, the ultradian rhythm orchestrates behavioural activity and enhances responsiveness to an external stimulus. This suggests a role for the circadian-ultradian timekeeping hierarchy in governing organized behaviour and shaping daily experience through coordinating the motivation to engage in recurring, albeit not highly predictable events, such as social interactions.

## Author summary

Biological rhythms allow animals to carry out behaviours at a suitable time, but ultradian rhythms with a period shorter than a day do not match the period of any cycles in the physical environment. They remain mysterious in both their biochemical mechanisms and their functional significance. Previous studies have demonstrated a link between striatal dopamine (DA) and ultradian behavioural rhythms. Based on mechano-biological findings of DA regulatory processes, we proposed a model where DA self-feedback through D2 autoreceptors generates ultradian oscillations. Using simulations, we were able to reproduce ultradian DA rhythms that resemble behavioural rhythms. We further explored how these rhythms respond to circadian regulation and external stimuli, the two major signals of behavioural relevance. The results show that the presence of ultradian rhythms within a functional circadian system offers organized daily behaviours, with increased flexibility and heightened responsiveness to significantly arousing experiences.

## Introduction

Biological rhythms are ubiquitous in the natural world. One functional significance of overt rhythmicity is to synchronize the host’s physiological and behavioural states with regular events in the environment. But unlike circadian rhythms that have a narrowly-defined period of approximately 24 hours corresponding to the solar day, ultradian rhythms are defined broadly as those with periods shorter than circadian and do not correspond to any known physical cycles (see reviews: [1–4]). In mammals, ultradian rhythms have been recorded in various physiological and behavioural activities including heart rate, respiratory rate, body temperature, wheel-running, horizontal movement, drinking, and eating. Amongst which, the ultradian period of individual locomotor and feeding rhythms tends to dynamically fluctuate around 3–5 hours when measured under both alternating light-darkness and constant conditions, displaying considerable flexibility at both the inter- and intra-individual levels [5–8]. This period flexibility, along with the inconsistent characteristics among consecutive cycles, has led to debates over whether these behavioural rhythms are generated by an ultradian oscillator or a result of stochastic episodic events [4, 9].

Just like their circadian counterpart, the ultradian behavioural rhythms observed in lab rodents retain their temporal structure in the absence of external time cues and after forced behaviour alterations. The locomotion and foraging rhythms of voles, mice, and rats continue under constant light and during food deprivation [6, 8, 10], while activity following rest deprivation retains the prior ultradian phase [6]. Locomotion in mice displays a strain-dependent frequency structure in the ultradian range, implying at least a partial genetic predisposition to the expression of ultradian rhythms [5]. These findings favour a hypothesis whereby a self-sustained oscillator, rather than a homeostatic regulator, coordinates these rhythmic behaviours. However, identifying the neural structures and biochemical reactions comprising the oscillator is necessary to support this hypothesis.

Desynchronization of individual circadian clocks has been suggested to give rise to ultradian rhythmicity, as ultradian behavioural rhythms are often superimposed on or masked by a circadian rhythm. Simultaneous elimination of both circadian and ultradian rhythms has occasionally been observed after lesion of the suprachiasmatic nucleus (SCN), the site of the primary circadian pacemaker [11, 12]. However, considerable studies also have documented persistent, if not actually enhanced, ultradian rhythms in animals rendered circadian-arrhythmic after SCN lesions [6, 7, 13, 14] or clock genes disruptions [8]. This evidence favours the independence of the ultradian oscillator from the circadian system. It is likely that the ultradian behavioural rhythms are generated and sustained by anatomically-distinct mechanisms, yet receive regulatory inputs from the circadian system and are thus partially affected by circadian disruption.

Another proposed candidate location for the ultradian oscillator lies within the dopamine (DA) system. In addition to a vital role in regulating voluntary activity and motivation, an intact DA system also displays rhythmicity ranging from single neuronal fast oscillations to a circadian rhythm in DA concentration [15, 16]. The circadian DA oscillations are believed to underlie several SCN-independent behavioural rhythms, including the methamphetamine-induced rhythmic locomotion [17, 18], the feeding-entrained food anticipatory activity [19–21], and the timestamped place-conditioned responses [22, 23]. A treatment targeting the DA system alone is sufficient to abolish rhythms with a regular ultradian period. Individual manipulations of DA transporters, DA receptors, and midbrain DAergic neurons all significantly alter the ultradian behavioural rhythm [8]. The most likely location of an ultradian DA oscillator is the mesostriatal DAergic neuronal projection, which comprises the majority of DAergic neurons in the central nervous system. In fact, striatal extracellular DA concentration displays an ultradian fluctuation that correlates with and precedes the ultradian fluctuations in spontaneous activity levels [8].

While the ultradian behavioural rhythms are likely attributed to the ultradian striatal DA rhythm, the mechanisms responsible for the generation of DA oscillations *per se* are more elusive. Brain DA concentration is under considerable regulation by both external factors and internal mechanisms [24]. Circadian rhythmicity has been observed for extracellular DA concentration [8, 25–27] but not in the DA level measured from microdissected brain regions [28]. This suggests that there are more complex regulatory mechanisms at work determining the intracellular and extracellular distribution of DA molecules. DA molecules are released into the extracellular space via exocytosis and are removed through reuptake by the presynaptic dopamine transporters (DAT). Both the firing activity of midbrain DAergic neurons and striatal DAT availability change throughout the day, bearing a partial but critical relationship with the circadian variation in extracellular DA [27, 29]. If extracellular DA can temporarily inhibit its own release and accelerate its own removal, then the DA self-regulation processes could potentially form negative feedback loops, which constitute the core mechanistic structure of biological oscillators.

Autoreceptors are pre-synaptic receptors that can regulate subsequent neuron activity after activation by ligands released from the neuron. The D2-type DA receptor is the dominant DA autoreceptor regulating striatal DA concentration (see review: [30, 31]). D2 agonists induced a similar effect as exogenous DA in suppressing neuron electrical activity [15, 29, 32, 33] and increasing DAT-dependent DA reuptake [34, 35], while D2 antagonists produced opposite effects [35, 36]. Co-administration of D2 antagonist can suppress the changes in release and removal rates induced by exogenous DA [37, 38]. Signal transduction via D2 activates a cascade of biochemical reactions which enhances potassium conductance [36, 39] and reduces calcium conductance [40, 41] at the nerve terminal. It also accelerates unidirectional translocation and cell surface expression of DAT [27, 35] while decelerating the phosphorylation and catalyzing capability of tyrosine hydroxylase, the rate-limiting enzyme of DA synthesis [42]. These downstream reactions ultimately decrease extracellular DA concentration [30, 31], but the extent to which these responses are normally activated is not documented. The presynaptic responses occur from seconds up to an hour after D2 activation and appear to be influenced by circadian timing, suggesting that D2 modifies DA oscillations on multiple time scales. However, the role of this striatal DA-D2 self-feedback loop in ultradian DA rhythm generation and ultradian behavioural rhythm expression has yet to be investigated.

Mathematical models have played a significant role in advancing our understanding of DA regulation and rhythmic dynamics. Best et al. [43] have meticulously modelled cellular DA processes to study DA dynamics involved in homeostasis. This model was then expanded by Kim and Reed [44] to incorporate circadian regulation on DA synthesis, degradation, and downstream signalling. Compared with circadian DA rhythm, the ultradian DA rhythm relates more to local mechanisms regulating DA release and reuptake and is less affected by the availability of DA cellular molecules [37]. A previous model of the hypothalamic-pituitary-adrenal axis [45] demonstrated the capacity of individual neural networks to autonomously produce ultradian rhythms through local processes involving negative feedback loops and respond to external inputs from the global circadian rhythm. It is thus possible that the ultradian rhythm observed in striatal DA tone is generated through local synaptic processes while modulated by the circadian system.

In this paper, we propose that a D2-mediated DA release-and-removal regulation generates ultradian oscillations in the extracellular DA concentration at the midbrain-striatum synapses, thereby driving the expression of ultradian behavioural rhythms. The following sections begin by illustrating the critical biochemical elements that form the modular concepts of the Dopamine Ultradian Synaptic Regulator (DUSR) model, described using ordinary differential equations and nominally based on data from nocturnal rodents, which are extensively studied in both the behavioural and biological context. After showing that the DA-D2 self-regulation can generate sustained ultradian oscillation, we proceed to investigate DUSR’s response to external inputs of behavioural relevance, which include an inhibitory circadian signal from the circadian system and transient excitatory stimulus corresponding to environmental events. The simulation results highlight the DUSR’s strong response to partially predictable external inputs comprising both a consistent circadian signal and a transient stimulus. Finally, we conclude with a discussion of model assumptions and the biological significance of DUSR and ultradian rhythms.

## Results

### Dopamine-D2 self-regulation produces an ultradian rhythm

**Fig 1:**
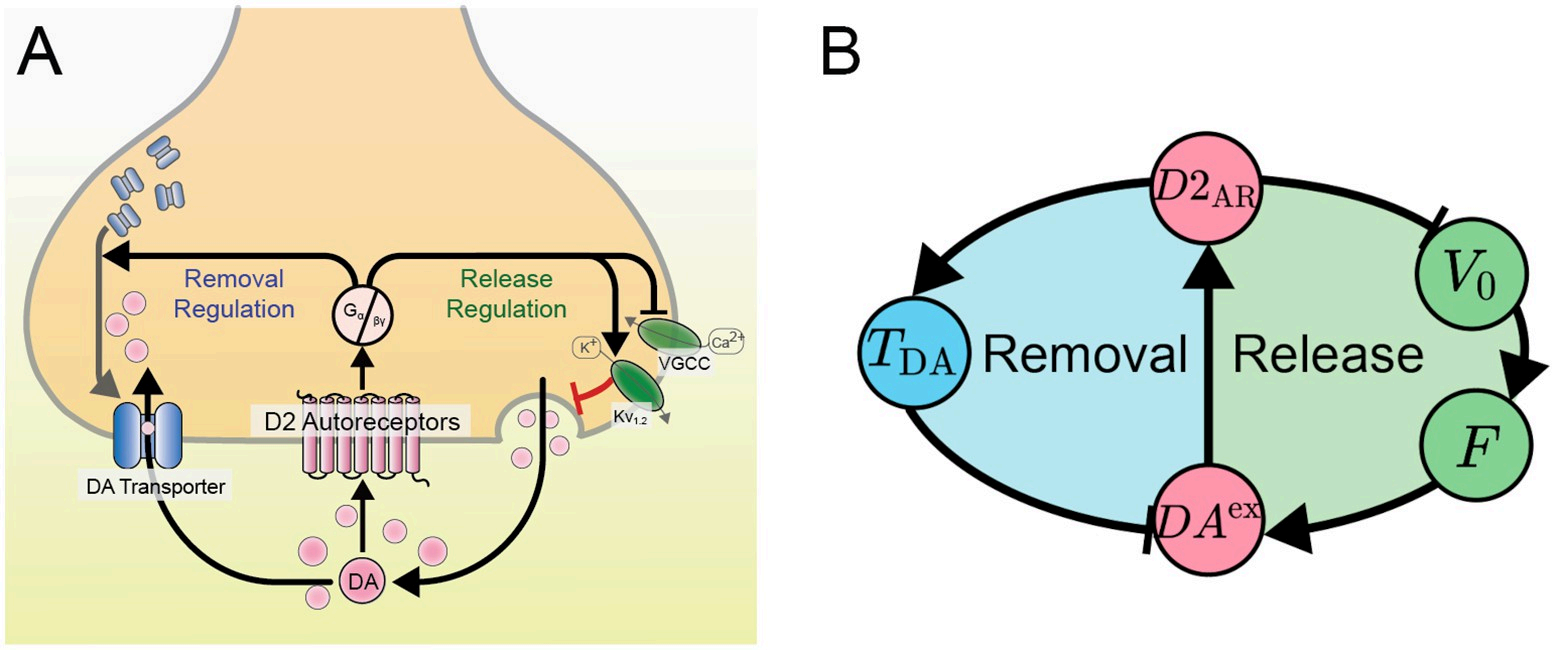
Biological processes and schematic representation of DA-D2 self-regulation. A: Striatal DA activates D2 autoreceptors, which triggers a reaction cascade through the G protein *G*_*α*/*β*_ that subsequently increases DA reuptake and decreases DA release. VGCC represents the voltage-gated calcium channels and *k*_*V*1_, *k*_*V*2_ represent the voltage-gated potassium channels. This picture is adapted from [30] B: Schematic diagram of the Dopamine Ultradian Synaptic Regulation (DUSR) model. Variables are defined in Table 1.

The biochemical processes critical to striatal extracellular dopamine (DA) self-regulation are depicted in Fig 1A, forming the foundation of the core Dopamine Ultradian Synaptic Regulator (DUSR) model. A schematic overview of the DUSR model is presented in Fig 1B, which shows the dynamic variables. Their corresponding biological meaning is listed in Table 1. The signalling of striatal extracellular dopamine (*DA*^ex^) at pre-synaptic D2 autoreceptors (*D*2_AR_) is marked by electrically-stimulated release and terminated by transporter-dependent removal. This presynaptic transmitter-receptor interaction forms the foundation of DA self-regulation. Our model thus incorporates a dual-negative feedback loop structure of DA release and DA removal respectively. Enhanced *D*2_AR_ signalling 1) increases dopamine transporter availability (*T*_DA_) and thus increases DA removal rate, as well as 2) reduces the average DAergic neuron membrane potential (*V*_0_), consequentially decreasing firing rate (*F*) and ultimately decreasing DA release rate. Further details on the relevant neurophysiology are given in the methods section, while the model statement is given below.

**Table 1:** Variables of the DUSR model.

*DA*^ex^ (μm)	Concentration of striatal extracellular dopamine
*D*2_AR_ (μm)	Concentration of activated pre-synaptic D2 autoreceptors
*V*_0_ (mV)	Average DAergic neuron resting membrane potential
*F* (Hz)	Average DAergic neuron firing rate
*T*_DA_ (1)	Pre-synaptic dopamine transporter availability

The state variables are *D*2_AR_, *V*_0_, and *T*_DA_, while *DA*^ex^ and *F* are determined instantaneously from the state variables.

The dynamic variables *D*2_AR_, *V*_0_, and *T*_DA_ evolve in time according to

$$\begin{array}{c} \frac{dD2_{\text{AR}}}{dt}=k\left(D2_{\text{tot}}-D2_{\text{AR}}\right)\left(DA^{\text{ex}}\right)-a\cdot D2_{\text{AR}}, \end{array}$$ (1)

$$\begin{array}{c} \frac{dV_{0}}{dt}=-c\cdot V_{0}+b\cdot F-k_{V}\cdot D2_{\text{AR}}, \end{array}$$ (2)

$$\begin{array}{c} \tau_{T}\frac{dT_{\text{DA}}}{dt}=1+\frac{\Delta T-1}{1+e^{-k_{T}\left(D2_{\text{AR}}-D_{0}\right)}}-T_{\text{DA}}. \end{array}$$ (3)

The variable *DA*^ex^ evolves according to

$$\begin{array}{c} \frac{dDA^{\text{ex}}}{dt}=\alpha \cdot F-\frac{k_{\text{Vmax}}\cdot T_{\text{DA}}}{K_{M}+DA^{\text{ex}}}DA^{\text{ex}}-\beta \cdot DA^{\text{ex}}. \end{array}$$ (4)

However, because *DA*^ex^ evolves on a faster timescale than the other variables, as has been observed in past measurements [46, 47] and apparent in the non-reduced model Eqs (1)–(4), its instantaneous value is determined through a quasi-equilibrium instead:

$$\begin{array}{c} DA^{\text{ex}}=\frac{\alpha F-\beta K_{M}-k_{\text{Vmax}}\cdot T_{\text{DA}}+\sqrt{{\left(\alpha F-\beta K_{M}-k_{\text{Vmax}}\cdot T_{\text{DA}}\right)}^{2}+4\beta \alpha F\cdot K_{M}}}{2\beta }. \end{array}$$ (5)

The variable *F* is determined by an instantaneous function of *V*_0_,

$$\begin{array}{c} F\left(V_{0}\right)=\frac{F_{\text{max}}}{1+\text{exp}\;\frac{\theta -V_{0}}{\sigma }}. \end{array}$$ (6)

The sigmoidal relationship between *F* and *V*_0_ introduces strong nonlinearity that impacts the evolution of *V*_0_ over time through self-regulation (second term of Eq (2)) and influences the determined concentration of *DA*^ex^ in Eqs (4) and (5).

All parameters of the model and their values are listed in Table 2 along with sources, if applicable. The parameter choices are discussed in detail in the methods section.

**Table 2:** The DUSR model parameters.

parameter		nominal value	references
DA release / reuptake / other processes			
*α*	DA unit release amount	0.09 μm	[48]
*k*_Vmax_	Baseline reuptake rate	2.63 × 3600 μm h^−1^	[48, 49]
*K*_M_	Michaelis-Menten constant of reuptake	0.2 μm	[48, 49]
*β*	Other DA processes	144 h^−1^	[50, 51]
DA-D2 interaction			
*D*2_tot_	Total D2	0.1 μm	[47]
*k*	DA diffusion rate × DA-D2 binding rate	10.46 μm^−1^ h^−1^	*
*a*	DA-D2 unbinding rate	1.7 h^−1^	*
Dopaminergic neuron resting membrane potential regulation			
*c*	Neuron restoring rate	3.62 h^−1^	[52]
*b*	Neuron-excitatory feedback rate	0.012 mV	*
*k*_V_	D2-induced hyperpolarization rate	2.73 × 3600 mV μm^−1^ h^−1^	*
Determination of firing rate			
*F*_max_	Maximum firing rate	15 × 3600 h^−1^	[53]
*θ*	Population average firing threshold	25 mV	[54–56]
*σ*	Population firing threshold variation	18 mV	*
DAT cytosol-membrane translocation			
Δ*T*	Maximum DAT availability	1.8	[35]
*D*_0_	Half efficiency of D2	0.04 μm	*
*k*_T_	Plasticity factor of D2 on DAT translocation	87.5 μm^−1^	*
*τ*_T_	Time delay of D2 regulation of DAT	0.15 h	[35]

References of the nominal values are listed when applicable. Decisions on parameter value choices are described in the methods section.

**Fig 2:**
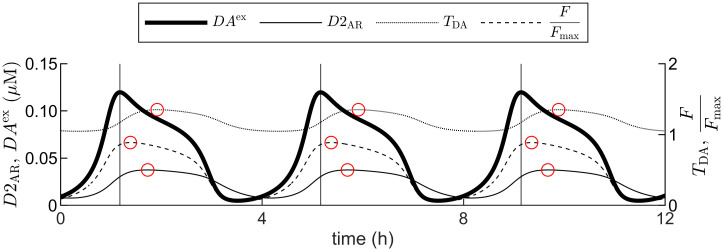
Simulated output of the core DUSR model. An ultradian rhythm (period = 4.0 h) for key output variables: extracellular dopamine (*DA*^ex^), activated D2-autoreceptors (*D*2_AR_), dopamine transporter activity (*T*_DA_), and DAergic neuron firing rate (*F*). *F* is plotted as a ratio over the maximum firing rate *F*_max_. Vertical lines indicate the peak phases of DA; red circles on the plotted lines mark the peak phase of the respective variable. *F* of the release loop phase-leads *D*2_AR_ and *T*_DA_ of the removal loop.

Upon simulation with the given parameter values and in the absence of further inputs, the core DUSR model generates a sustained oscillation with a period of 4.0 h (Fig 2). The oscillation in our model aligns with the principles of biochemical oscillators [57]. While the core requirement of a negative feedback loop is satisfied by the model’s dual-negative loop structure, it alone does not ensure oscillations. It is also essential that each step in the feedback loop introduces a similar amount of signal distortion with adequate delay [58, 59]. The delay in our model is achieved implicitly within a minimal 3-stage loop with quasi-steady-state assumptions. This structure could readily give rise to oscillations upon appropriate choices of reaction rates [57, 60, 61]. Nonlinearity is present in both feedback loops through *T*_DA_ and *F*; each equation is balanced between the opposing processes; and the positive self-feedback in Eq (2) amplifies deviation. Together, these factors create a conducive environment for oscillation production. The given parameter set in Table 2 is tuned to satisfy nonlinearity and delay requirements, leading to stable oscillations. The parameter dependence of this ultradian oscillation is reported in the following section.

We report the following quantitative outputs of our model during stable oscillation along the limit cycle: 1) Striatal extracellular DA concentration *DA*^ex^ oscillates between 4.9 nm and 120 nm around a mean value of 56 nm, remaining in the reported lower nanomolar range [8]; 2) D2 autoreceptor occupancy *D*2_AR_ oscillates between 7.8 nm to 37.6 nm with a mean value of 24 nm, conforming to the lower-tenth of baseline striatal D2 occupancy [30, 62, 63]; 3) Transporter availability *T*_DA_ of the removal feedback loop oscillates from approximately 87% to approximately 115% of the average 1.2 throughout the cycle; 4) Midbrain DAergic firing rate *F* of the release feedback loop oscillates between 0.8 and 13.3 Hz with an average value of 7.2 Hz; 5) The phase differences between *D*2_AR_, *T*_DA_, and *F* with *DA*^ex^ are 0.53 h, 0.74 h, and 0.21 h respectively. The coordinated oscillation of both feedback loops at 4.0 h suggests that the structural organization of DA-D2 self-regulation is predisposed to joint oscillation.

### The DUSR oscillation is flexible and sensitive to parameter choices

**Fig 3:**
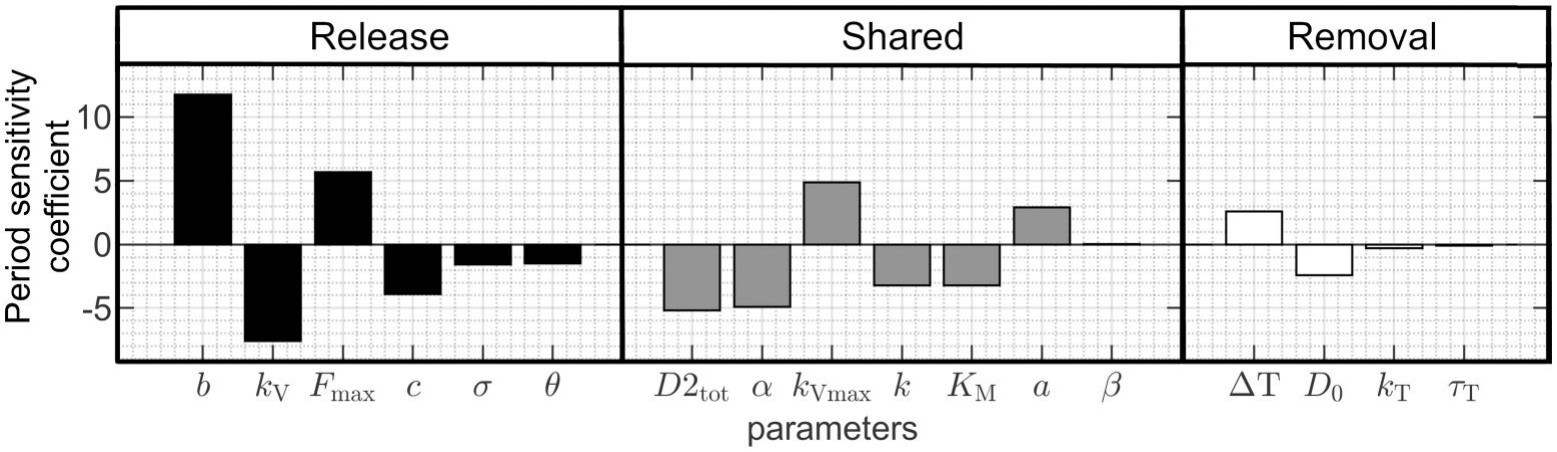
Local sensitivity of the simulated ultradian period. Local period sensitivity analysis performed at the nominal period of 4.0 h using Eq (7); Nominal parameter values listed in Table 2. The period is in general most sensitive to parameters unique to the release loop, moderately sensitive to parameters shared by both feedback loops, less sensitive to parameters unique to the removal loop, and not sensitive to parameters not directly involved in DA feedback (*β*).

At the nominal period of 4.0 h, the oscillation period of the simulated ultradian rhythm is sensitive to individual perturbations to most of the 17 model parameters, as shown in the results of the local sensitivity analysis (Fig 3). The model displays the highest local period sensitivity to parameter *b* governing excitatory neural regulations, for which a 1% change to the parameter produces twelve times a change in the output period (12%). The period is most sensitive to the parameters related to DAergic neuron activity (*b*, *k*_V_, *F*_max_) and is moderately sensitive to most parameters involved in both regulatory feedback loops, including those determining D2 occupancy (*D*2_tot_, *k*, *a*) and the instantaneous DA concentration (*α*, *k*_Vmax_, *K*_M_). The model shows less sensitivity to parameters unique to the removal feedback loop defining the D2-regulated availability of DAT (Δ*T*, *D*_0_, *k*_T_, *τ*_T_). Additionally, the period is relatively insensitive to the parameters (*θ* and *σ*) relating *F* to *V*_0_ in Eq (6). Alongside the period’s high sensitivity to parameters regulating DA membrane potential, this justifies the direct computation of neural firing activity from neural electrical properties, supporting the role of D2-regulation on neuron excitability in ultradian oscillation production. Finally, parameter *β*, which is not directly part of the DA feedback loops, has minimal effect on the period due to its lack of direct relationship with the output oscillation. The sensitivity coefficients of the DUSR rhythm’s period at 4.0 h averages to 3.6 for the entire parameter set, which could be considered highly flexible when compared to the circadian and other biological rhythms expected to remain in phase with a precise physical cycle. Despite their biological correspondence to involved pathways, some parameters with less impact on the period may be reduced while preserving ultradian oscillations to increase model simplicity. This will be considered in future works and may further enhance the overall flexibility of the ultradian rhythm to internal variations.

**Fig 4:**
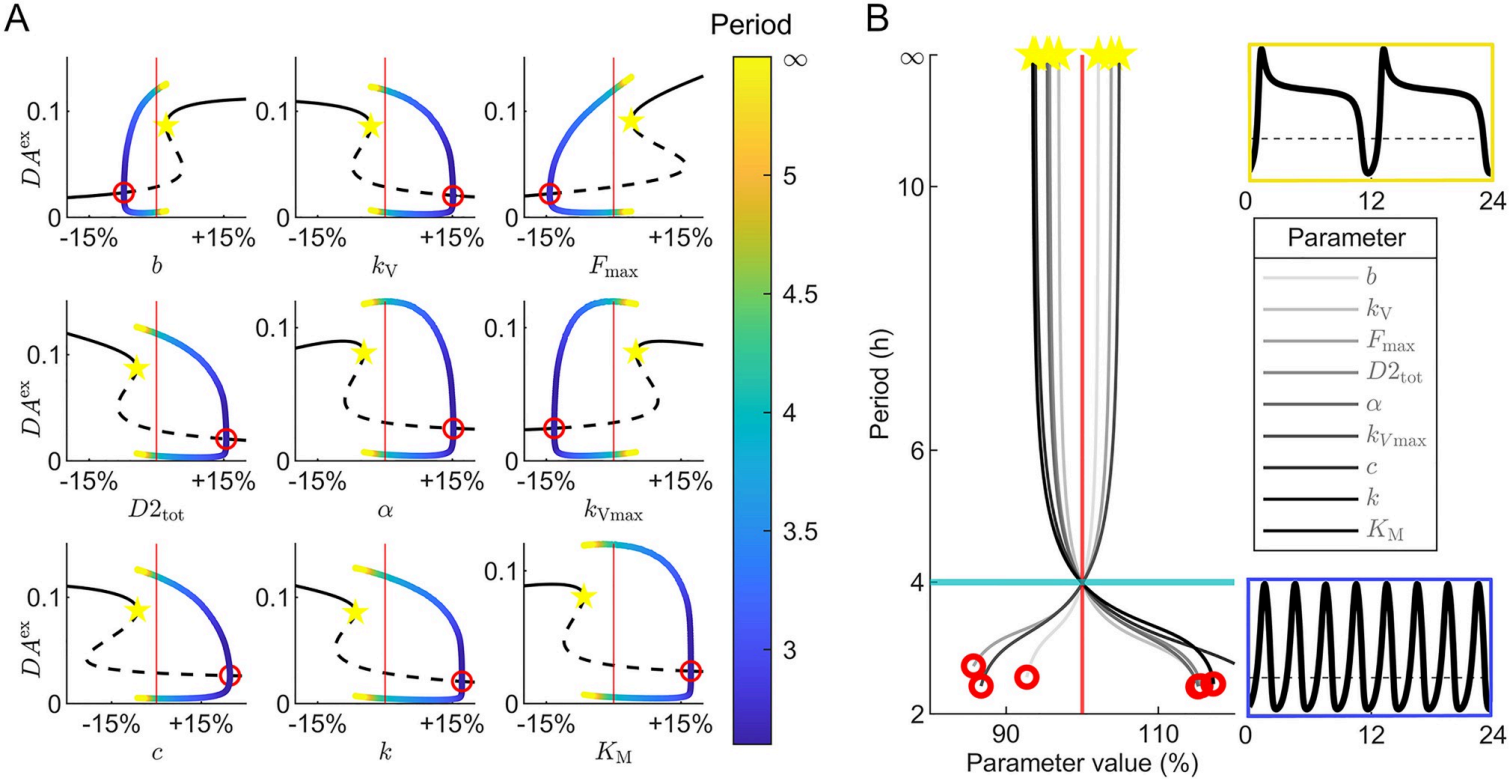
Bifurcation analysis of the core DUSR model. A: Bifurcation diagrams for nine parameters with top local period sensitivity. Black curves indicate the stable (solid) and unstable (dotted) steady states for *DA*^ex^. Coloured curves identify the limit-cycle oscillatory behaviours (max, min of the oscillation; period in colours). Stable DA equilibrium is replaced with self-sustained oscillations marked at the supercritical Hopf bifurcation point (red circle) and re-emerges from a saddle-node bifurcation (yellow star). An infinite period solution appears at the moment of the saddle-node bifurcation, forming a saddle-node on invariant cycle (SNIC) bifurcation and is characterized by heightened sensitivity of both the presence and the period of the DA oscillation to parameter values. B: The corresponding ultradian period at parameter values within the stable limit-cycle range. The majority of the period flexibility occurs within ±2 h of the nominal period (4.0 h) when shifting individual parameters within their oscillatory range. *DA*^ex^ timecourses for a long period (12 h) and a short period (3 h) are shown above and below the parameter legend.

To further explore the range and limits of the DUSR-sustained ultradian oscillations, bifurcation analysis was performed for each of the nine parameters with the highest local period sensitivity as shown in Fig 3. Starting from the nominal parameter value given in Table 2, the qualitative behaviour of *DA*^ex^ at equilibrium and during limit cycle oscillations is plotted beyond the oscillatory range in both directions (Fig 4A). These nine components are essential for ultradian oscillation, such that oscillations are lost when a parameter is shifted beyond around 5–20% from its nominal value. For each parameter, we observed that the ultradian DA oscillation emerges with a period of roughly 2.5 h and an amplitude of zero at a supercritical Hopf bifurcation. The oscillation disappears at a saddle node on an invariant circle (SNIC) bifurcation [64]. As the parameter value approaches the vicinity of the SNIC bifurcation point, the period tends toward infinity. The complex bifurcation behaviour of the DUSR system suggests that it is possible to substantially extend the ultradian oscillation period through parameter modifications, whereas decreasing the period is constrained by a lower threshold. However, when shifting an individual parameter within its oscillatory range, the majority of ultradian period changes fall within ± 2 hours (Fig 4B) before losing rhythmicity.

The presence of multiple bifurcation types further diminishes the robustness of the ultradian rhythm against perturbations, enabling the DUSR to generate a spectrum of continuous outputs with abrupt changes in DA behaviour in response to internal environmental changes taking the form of parametric alterations. The system’s oscillatory behaviour becomes highly sensitive to parameter values near the SNIC bifurcation point, resulting in considerable increases in period length or the complete disappearance/emergence of oscillations. Around this point, the prolonged oscillation period is associated with a substantial increase in equilibrium DA levels. While the parameter range for sustained oscillations may initially appear as limited, the flexibility and unpredictability of the ultradian oscillator can lead to the sporadic ultradian phase and period changes observed in animal behavioural recordings as well as the inter-individual difference in striatal DA level [7, 8]. Furthermore, external inputs can induce the ultradian oscillator to exhibit ultradian rhythms beyond its typical oscillatory range, as demonstrated in the following section.

### Circadian rhythm bidirectionally modifies the ultradian rhythm and consolidates behaviour towards the early active phase

**Fig 5:**
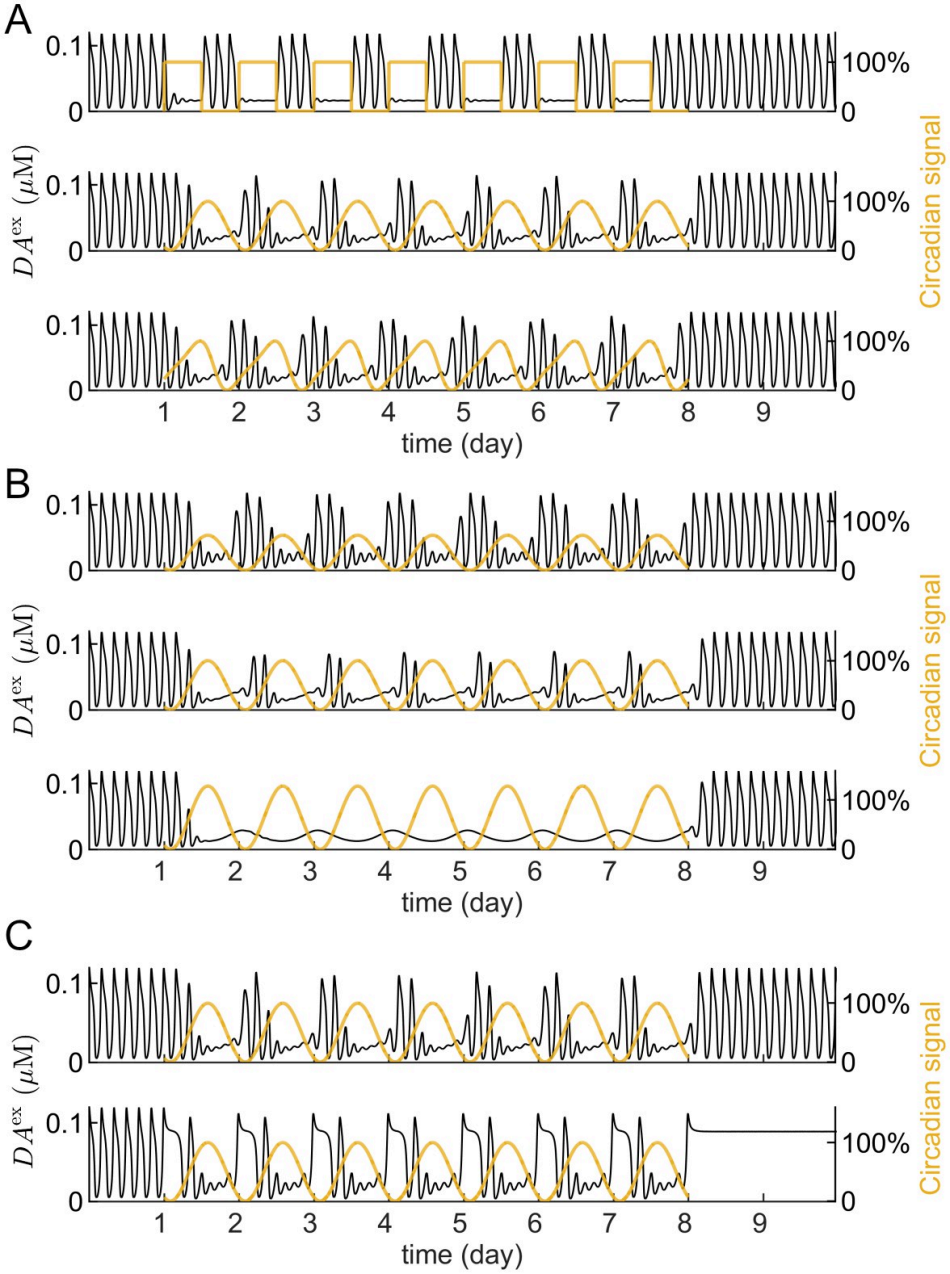
Circadian regulation on the ultradian rhythm. The effect of an inhibitory circadian-signal input (yellow line) *R*_circ_ to *V*_0_ on extracellular dopamine concentration *DA*^ex^ (black line) over time. A: Circadian inputs of different waveforms consolidate ultradian DA episodes within a circadian active phase. B: Increasing the circadian signal’s strength substitutes its consolidating effect on the ultradian rhythm with a complete masking effect. C: Circadian-signal modification has a bidirectional effect on the ultradian rhythm. Depending on the DUSR system’s intrinsic oscillatory state, removal of circadian regulation results in either a more pronounced ultradian rhythm (upper panel) or the loss of quasi-ultradian episodes (lower panel).

We choose to study the circadian system’s modulatory impact on the DUSR system by introducing an inhibitory signal that acts on *V*_0_ of the release loop. This inhibitory signal is aligned with the antiphase relationship between the SCN and behaviour in nocturnal rodents [65, 66], which we outline in the methods section ‘Effects of circadian inhibitory inputs on DA oscillations’. The addition of this circadian input results in *DA*^ex^ displaying an intrinsic ultradian oscillation superimposed on a forced circadian oscillation (Fig 5A). Circadian-signal inputs shape the ultradian rhythm to exhibit daily alterations between pronounced and suppressed activity. Despite high inter-individual differences, striatal DA concentration exhibits a phasic relationship with behaviour episodes, where local increases in DA concentration precede heightened behavioural activity [8]. Consequently, the alterations in the amplitude of ultradian *DA*^ex^ oscillations could correspond to the behaviourally active and inactive phase. A block-wave circadian signal generates three distinct ultradian episodes during the active phase, while continuous sinusoidal circadian inputs generate two or three ultradian episodes during the active phase, with minor episodes extending into the inactive phase. As the amplitude of the circadian input increases, its consolidating effect on the ultradian rhythm transitions into a masking effect, where the forced circadian rhythm completely replaces the self-sustained ultradian rhythm (Fig 5B).

Depending on the DUSR system’s oscillatory state, the circadian-signal input can either mask or stimulate the ultradian oscillation (Fig 5C). When the system exhibits a stable limit cycle, an inhibitory circadian signal input has a sole masking effect whose strength increases with the signal amplitude. Upon suspension of the circadian signal, the ultradian rhythm gradually resurfaces with increasing amplitude. When the system exhibits a stable equilibrium (Fig 5C, bottom plot. *k*_V_ changed to 2.64 × 3600 mV μm^−1^ h^−1^; other parameter values remain as listed in Table 2), the non-oscillatory DUSR system can be induced to exhibit quasi-ultradian oscillation with a moderate-strength circadian-signal input as the model becomes a forced oscillator constantly pushed away from its equilibrium. In this case, removing the circadian input results in DA gradually returning to its stable equilibrium, where both the circadian and ultradian rhythms are lost. This finding that removing the circadian input leads to either increased ultradian rhythms or complete arrhythmicity potentially reconciles previous contradictory studies, which report either a complete loss or preservation of ultradian rhythms post-SCN lesion [6–8, 11–14].

**Fig 6:**
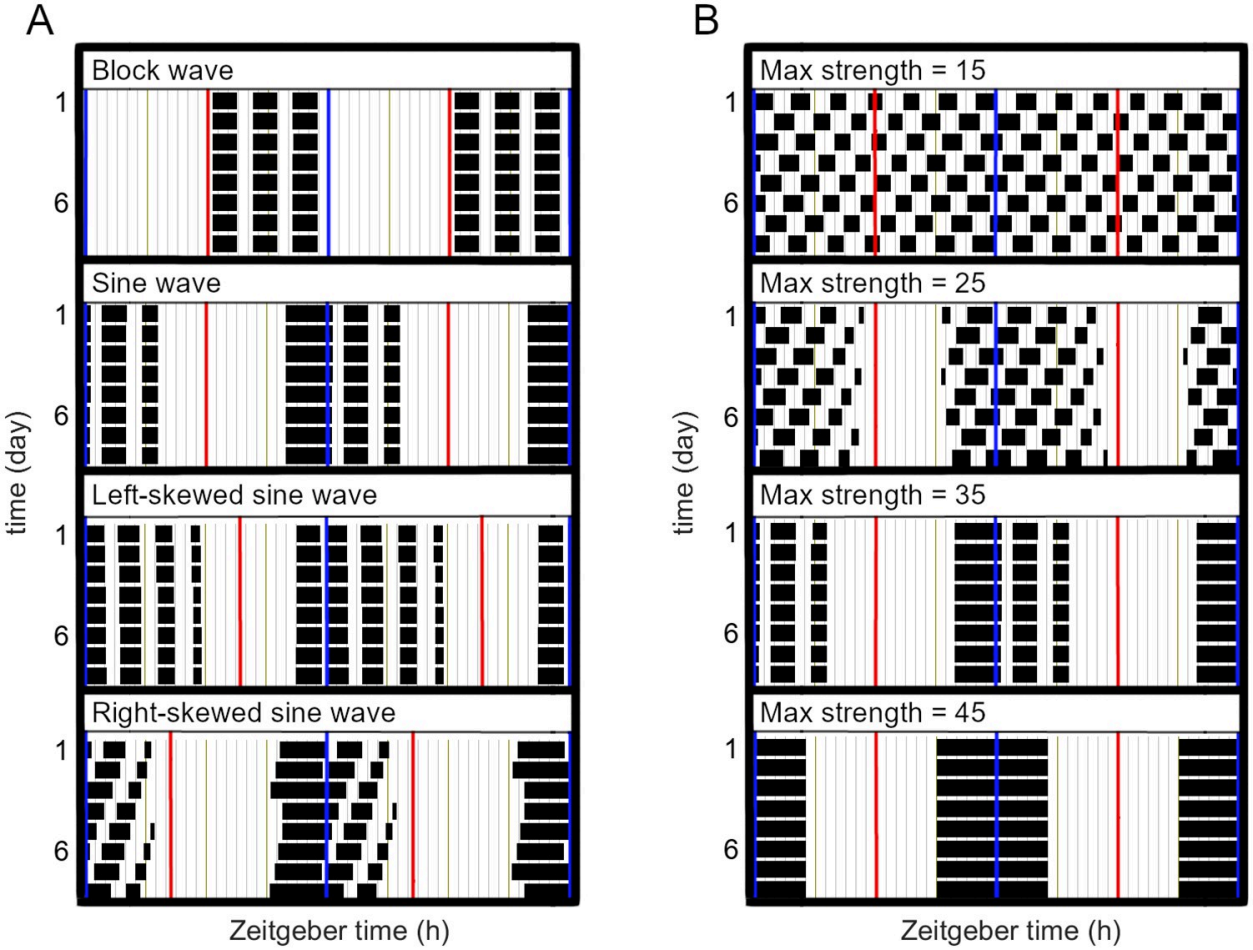
Behavioural activity output of the DUSR under circadian regulation. Double-plotted activity defined as when *DA*^ex^ values exceed the previous day’s average, Eq (11). A: Behaviour activity for circadian signals of different waveforms and same amplitude. Red lines mark the peak circadian input and blue lines mark the trough. B: Behaviour activity output for increasing amplitudes of a symmetric sinusoidal circadian signal is increasingly refined within the subjective night.

The behavioural activity output of the model (Fig 6), derived from simulated DA dynamics using a circadian moving average method detailed in the section ‘DA oscillations and behavioural activity’, shows the impact of both circadian strength and waveform on daily behavioural activity pattern. When subjected to moderate circadian input, the model generates behavioural patterns characterized by distinct circadian and ultradian components (Fig 6A). A block-wave circadian signal, with abrupt transitions between states, produces three clearly defined ultradian bouts of approximately equal duration, all occurring within the off phase of circadian inhibition. In contrast, sinusoidal circadian signals with smoother transitions produce behavioural active phases that encompass the trough of circadian modification and generate daily ultradian behavioural patterns consisting of a long episode followed by shorter bouts. This corresponds with the SCN electrical and secretion activity in nocturnal rodents, which peaks several hours before locomotor activity onset and remains low during the subjective night [67]. It also aligns with the behaviours of nocturnal rodents in laboratory conditions, which tend to be confined within the subjective night and more concentrated towards the earlier night [7, 68]. These alignments suggest that a smooth-transit circadian signal resembling the waveform of SCN electrical activity better represents the circadian modulation received by the mesostriatal dopamine system [65].

Varying the circadian signal strength on the DUSR system leads to a spectrum of behavioural activity patterns (Fig 6B). A circadian signal with high amplitude confines all activity bouts within the subjective active phase of the day. Reducing the amplitude of the circadian signal results in more diffused activity bouts that extend into the subjective day, still superimposed upon a weak but significant circadian pattern. When the circadian signal weakens further, ultradian activity bouts become increasingly evenly distributed. These diverse patterns closely mirror behavioural changes in nocturnal rodents after partial and complete SCN lesions, where the night-only behavioural activity becomes more heterogeneously and homogeneously distributed throughout the entire circadian day [7]. This highlights the possibility of a common framework of varying circadian-ultradian coupling strength organizing the daily temporal structure of activity.

### Transient arousing inputs have a phase-dependent effect on the oscillation properties

**Fig 7:**
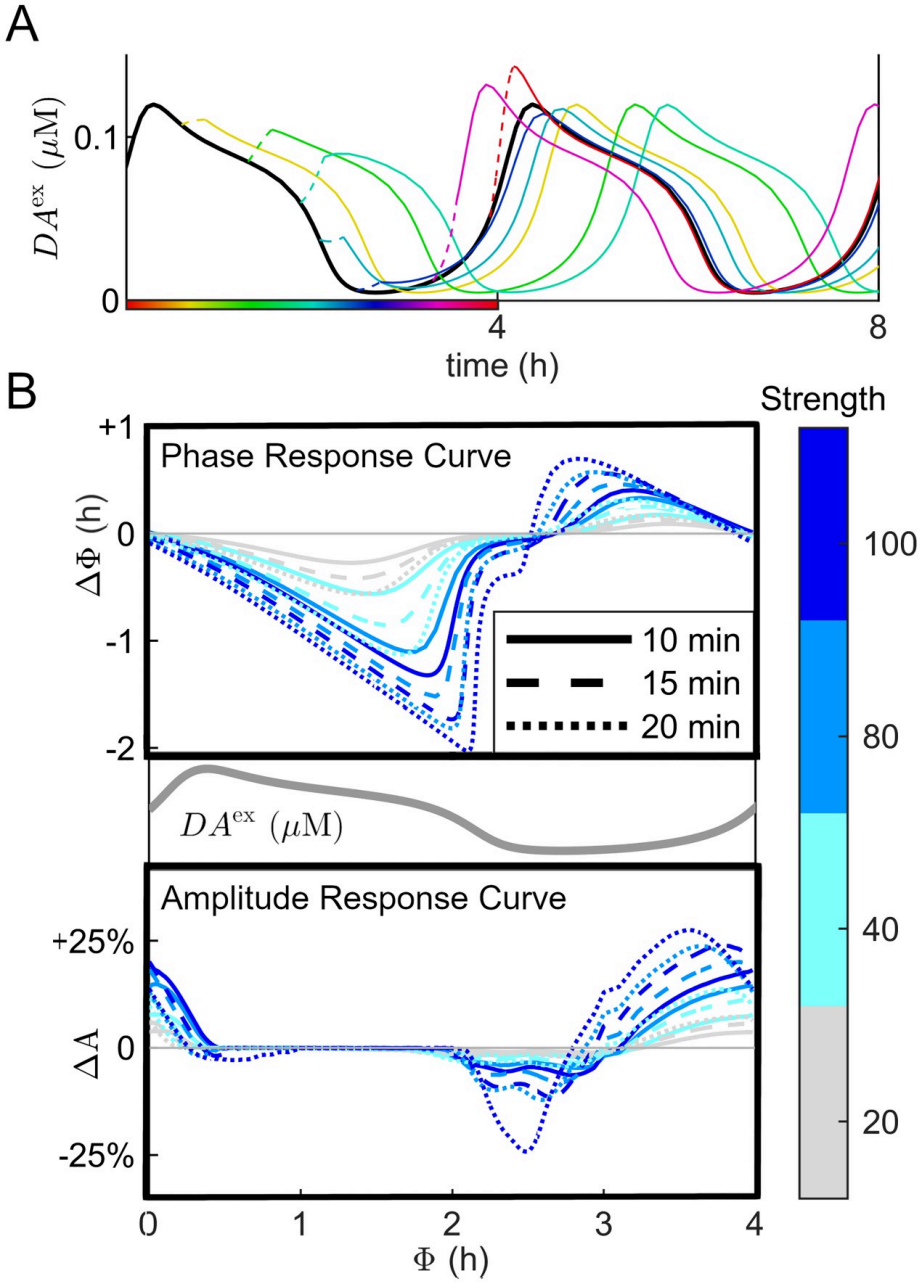
Phase-dependent effect of a transient excitatory pulse on the ultradian oscillation. A: *DA*^ex^ behaviour in response to a single pulse input (*I*_sens_ = 75, duration = 15 min) given at various phases. The black solid line represents the *DA*^ex^ trajectory without additional input. The dotted and solid coloured lines represent *DA*^ex^ trajectories during and after the pulse, with colours denoting the ultradian phase when the pulse is given. B: Phase response curve and amplitude response curve of *DA*^ex^ to a pulse input of various magnitude/duration combinations. The evoked phase shift and the percentage change of maximum-minimum *DA*^ex^ values within the immediate cycle are plotted against the timing of a centred pulse on the ultradian cycle.

We also choose to study the impact of a transient arousing experience simulated as a single excitatory pulse to *V*_0_. This pulse reflects the selective excitatory inputs to DAergic neurons associated with the subjective perception of a significant event [69, 70], which we explain in the methods section ‘Effects of transient excitatory inputs on DA oscillations’. This input has a phase-dependent effect on both the phase and the amplitude of the ultradian DA oscillation (Fig 7). An identical input could result in a spectrum of outcomes, including an advance or delay to the DUSR oscillation while temporarily strengthening or suppressing its amplitude. The duration of the pulse’s effect on DA trajectory also varies with phase, typically lasting for no more than two ultradian cycles.

The phase response curve (PRC) of *DA*^ex^ to a single excitatory pulse is characterized by an unimodal curve intersecting the x-axis slightly preceding the peak and the trough (Fig 7B). A pulse received during the DA rising phase advances the DA rhythm and one during the falling phase delays it. The corresponding amplitude response curve (ARC), determined as the transitory difference in the maximum and minimum *DA*^ex^ values within a subsequent cycle post-input, intersects the x-axis following the peak and the trough. It remains around zero during the semi-plateau phase after the DA peak. Specifically, the ultradian DA oscillation’s amplitude is augmented by the input during the rising phase, unaffected during the semi-plateau, and slightly suppressed during the rapid falling phase. Increasing the strength and duration of the input pulse broadens the augmenting range of the ARC, while skewing the PRC from a smooth transition into a sawtooth curve (Fig 7B; deep blue, dotted line).

The strength and the duration of the stimulus are reciprocally related. Short, strong inputs yield similar effects as long, milder inputs with equal products in magnitude and duration (e.g., the 40 strength-20 min pulse and the 80 strength-10 min pulse), producing primarily overlapping response curves. In the absence of other rhythms, a transient arousing input received before a local peak advances and enhances the upcoming ultradian episode, while one received during or after a high DA episode prolongs the current episode and delays the subsequent one without affecting its strength. The reciprocity between intensity and duration in ultradian responses to arousal mirrors that seen in circadian responses to photic inputs. The extent of this reciprocal effect depends on the cumulative strength between the stimulus’s perceived valence received by the mesostriatal DA system and its seemingly prolonged duration in minutes, which aligns with the organism’s need for a clock system responsive to potentially significant and recurrent events while filtering out responses to minor stimuli.

### Circadian-nested ultradian oscillators integrate and amplify external stimuli

**Fig 8:**
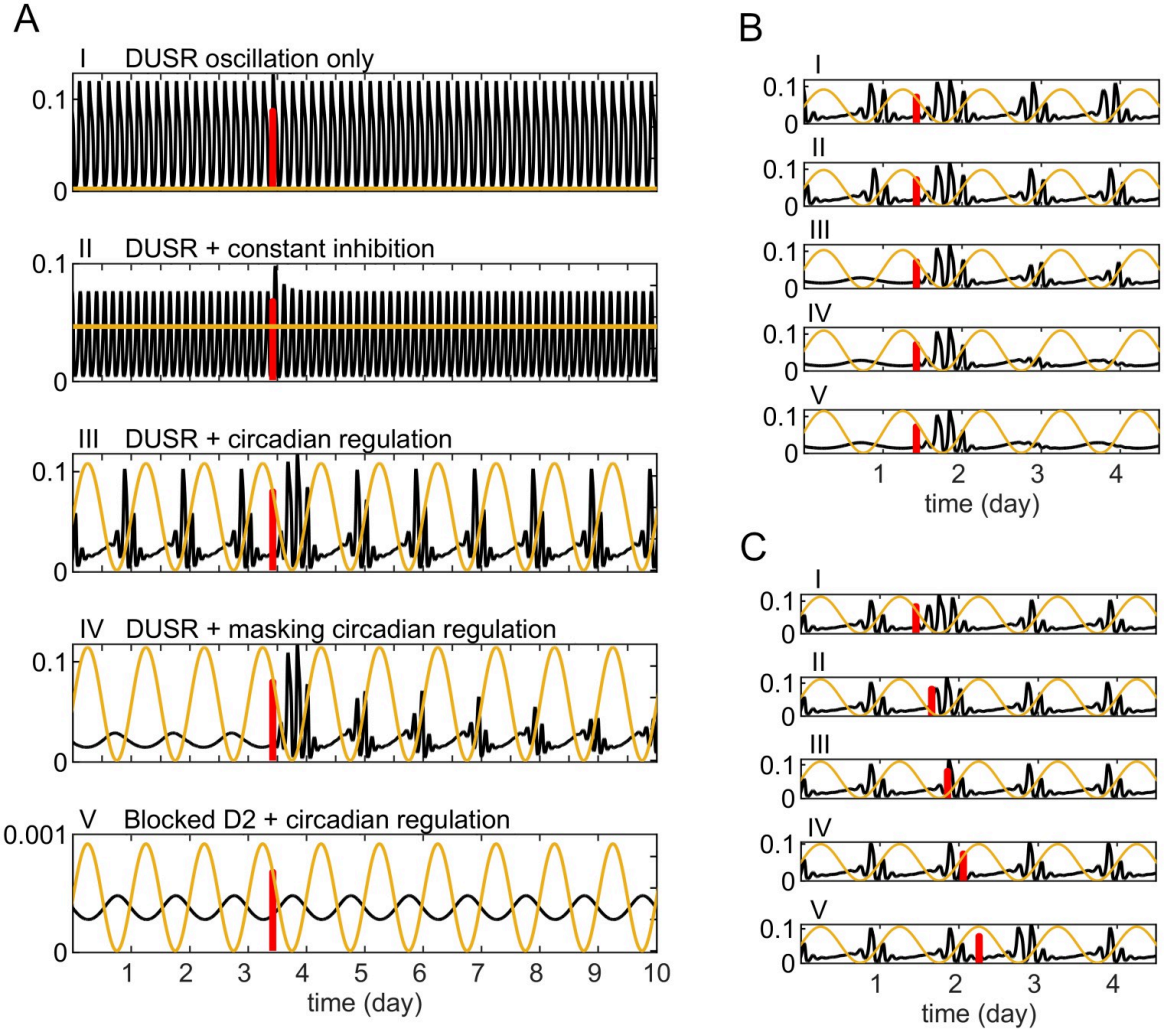
Circadian-ultradian interaction enhances responsiveness to transient stimulus. Simulated *DA*^ex^ trajectories (black line) of the DUSR model with an inhibitory circadian signal (yellow curve) and a transient excitatory pulse (vertical red line). A: The effect of a transient excitatory pulse on the DUSR model is amplified by the circadian inhibitory signal. Plotted from top to bottom are the *DA*^ex^ response trajectories to (I) an excitatory pulse of the DUSR model, (II) DUSR with a constant inhibitory signal, (III) with a circadian inhibitory signal, (IV) with a masking circadian inhibitory signal, (V) with a circadian inhibitory signal and the ultradian DA feedback loop fixed so that *D*2_AR_ and *T*_DA_ are held constant and *DA*^ex^ decreases in scale. B: Effect of an identical transient excitatory pulse given at the same phase of on *DA*^ex^ under increasing circadian regulation. C: Effect of an identical transient excitatory pulse given at different circadian phases on *DA*^ex^.

Both an inhibitory circadian signal and a transient excitatory input are applied to *V*_0_, simulating the response of striatal DA to external arousing experiences within a circadian context. In comparison to the response observed in the absence of circadian modification as reported in the previous subsection, the effect of an excitatory pulse is now significantly dependent on the circadian phase (Fig 8C). Notably, excitatory pulses administered around the trough of the circadian signal have an immediate augmenting effect on the ultradian DA rhythm. Conversely, pulses given around the acrophase of circadian inhibition produce a delayed stimulating effect that could be observed during the subsequent DA active phase (Fig 8C, plot V). Both the magnitude and the duration of response are now also prolonged to a circadian time course. This effect is particularly evident when the circadian signal exerts a masking effect on the ultradian rhythm (Fig 8B).

Fig 8A offers a comprehensive comparison of typical DA response trajectories to a transient stimulus under various background conditions: I) the DUSR ultradian oscillation alone, II) the DUSR under constant inhibition, III) the DUSR with normal inhibitory circadian regulation, IV) the DUSR with strong, masking circadian regulation, and V) the DUSR with feedback loops sequestered at *D*2_AR_ and with normal circadian inhibition regulation. In the absence of a circadian signal (Fig 8A, I and II), a transient stimulus slightly alters the amplitude of approximately one ultradian cycle and DUSR returns to its limit cycle oscillation quickly. In the absence of the ultradian oscillation (Fig 8A, plot V), the inhibitory circadian signal suppresses extracellular DA concentration to a low level and a transient stimulus induces a brief increase in DA that is quickly reversed. In contrast, DUSR with circadian regulation (Fig 8A, III and IV) showcases the largest relative change in amplitude, coupled with a prolonged response duration. This behaviour likely arises from the circadian signal, an external oscillation of a different frequency, consistently perturbing the DUSR oscillation away from its limit cycle. Consequently, DUSR becomes both a forced oscillator at the circadian frequency and an oscillator at its inherent ultradian frequency. This dual-frequency resonant behaviour necessitates an extended recovery time after perturbations, resulting in a prolonged response observed in DA concentration. The DUSR system, in this context, functions as a resonator for temporal inputs from the circadian system in addition to being a self-sustained oscillator of its own. This unique property enables external arousing events to induce amplified responses in striatal DA. The nested circadian-ultradian structure of the striatal DUSR could thus function as a local amplifier, organizing and amplifying subjective responsiveness to external stimuli throughout the day.

## Discussion

The current understanding of ultradian behavioural rhythms is limited by challenges in identifying, isolating, and directly manipulating the underlying oscillator. Our initial goal was to model an oscillation in striatal extracellular DA concentration known to correlate with ultradian behavioural episodes. Our mathematical model mechanistically links established DA self-regulatory processes to the generation of ultradian oscillations. Striatal extracellular DA concentration is both an output signal and a key component of the dopamine ultradian synaptic regulator (DUSR) and is driven by dual autoreceptor-dependent regulatory loops of DA release and removal. To focus on extracellular DA dynamics, cytosolic processes were simplified using standard form kinetics that best capture the dynamics of DA release and DA removal. By simulating the DUSR under different conditions, we observed flexible ultradian oscillations with an adjustable period displaying diverse responses to the synergistic interactions between a circadian inhibitory signal and a transient excitatory stimulus. We discuss the biological implications of the results, focusing on the functional role of a circadian-ultradian timekeeping hierarchy in organizing the temporal structures of subjective responsiveness.

### Assumptions and limitations of the model

The regulation of extracellular dopamine concentration in the striatum is a complex process. Our model simplifies presynaptic cytosolic processes of both feedback loops due to a focus on extracellular signalling.

#### DA Release feedback loop

The release rate determines the increase in DA concentration *DA*^ex^ with a linear dependence on the neuron firing rate *F* that represents the averaged population behaviour. The model thus does not differentiate between burst and tonic firing patterns, only using them as limiting factors for parameterization. While firing patterns are believed to play a key role in single-neuron fast oscillations, there lacks substantial evidence to support a relationship between firing patterns and longer rhythms.

Eqs (2), (4) and (6) capture the reduction in DA release by impulse-regulation on neuron excitability and the quantal release size is denoted by a constant parameter *α* in the model. D2 can also affect DA release by regulating DA synthesis through the inhibition of tyrosine hydroxylase, the rate-limiting enzyme that produces the DA precursor L-DOPA [42, 71]. However, administering exogenous L-DOPA did not replicate the effects on D2-dependent DAT upregulation as seen in administering exogenous DA [37]. This suggests that DA synthesis alone is insufficient to alter the DA feedback loops. This process is probably regulated to accompany the storage demand upon altering neuron activity and could be of more importance in extreme conditions, such as DA storage depletion after a psychostimulant overdose.

#### DA removal feedback loop

For the removal feedback loop, we modelled DAT-dependent reuptake using slightly modified Michaelis-Menten kinetics to account for a linear relationship between the maximum reuptake rate and the availability of DAT. The dynamics in D2-regulated DAT availability were modelled phenomenologically in Eq (3), focusing on the final consequences in DAT availability rather than the biochemical reactions. DAT availability is represented as a state variable that gradually approaches a time-varying final value determined by D2 activation. This approach aligns with the current state of knowledge on D2 accelerating DAT trafficking and re-balancing the dynamic equilibrium. While deletion and inhibition studies suggest that the process involves a signalling cascade of protein kinase C*β* and subsequently extracellular signal-regulated kinase (ERK) activation [34, 35], the complete regulatory pathway remains to be fully characterized and lacks quantitative information. The alternate hypothesis on the potential D2-DAT regulatory mechanisms suggesting direct D2-DAT interaction affecting individual DAT activity [72] was also excluded from the model due to a lack of solid supporting evidence under regular conditions.

#### Spatial homogeneity of DA and D2

Focused on rhythmic outputs, our approach prioritizes DA’s temporal dynamics over its spatial variability. We opted for ordinary differential equations (ODEs) over delayed differential equations (DDEs) in our model because all processes occur in close proximity and are based on rapid neurotransmitter actions. To simplify the analysis and allow for easier interpretation of the simulated results, we also assume spatial homogeneity of DA molecules and D2 autoreceptors in our model, representing the entire striatal extracellular space and presynaptic membranes as a single compartment using single variables. In reality, striatal D2 autoreceptors at different subcellular locations differ in their likelihood of being activated and contribute unequally to presynaptic DA regulation. Clusters of D2 autoreceptors can be found near active synapses, as well as on somas and unmyelinated axons distant from release sites [73–75]. While it is generally accepted that synaptically released DA activates extra-synaptic D2 autoreceptors through overflow and volume transmission, the subcellular localization of D2 relative to DA release sites may impact measurements of DA-D2 binding kinetics and interpretations of D2-DA regulation.

Autoreceptors adjacent to DA release sites attend to firing patterns and finely regulate DA [76]. Conversely, distant D2 autoreceptors likely respond to tonic DA signalling and DA overflow over longer timescales. Adjacent D2 autoreceptors are well-suited for exerting quick responses responsible for rapid oscillations at the single-neuron level, while distant D2 may contribute to longer rhythms in striatal extracellular DA concentration, such as the ultradian DA oscillations. Subcellular D2 localization may also contribute to the presynaptic regulatory effect of extracellular DA. Downstream processes of the D2 signalling cascade are proposed as limiting factor of D2 regulation [30] and exhibit sensitivities to subcellular location. Regulation of neural excitability by D2 is accomplished through regulating ion channels and would appear more significant near DA release sites compared to distant unmyelinated axons. The cytosolic regulation of DAT availability would be more consistent regardless of D2’s subcellular location, leading to the reuptake feedback loop responding to striatal background DA at a slower timescale.

In summary, our model simplifies by representing D2 and DA as single compartments responding instantly to concentration changes. Despite this, our model provides a framework for investigating more complex DA-related interactions. Future modifications of the model may incorporate spatial distributions to explore the interlinked relationship between distant D2 regulation, DAT regulation, and long rhythms in striatal DA tone. Determining whether subcellular D2 location significantly impacts DA regulation, particularly DAT regulation, will provide insights into the coordination of various rhythms. Considering the specificity of DAT translocation to D2 regulation and the dynamic nature of neural activity regulation [77], the removal loop may play a central role in establishing inherent ultradian rhythms, while the release loop could serve as a hub for integrating external information.

#### Inputs and outputs to the core oscillator

Our results demonstrate the potential of the DA negative feedback loops to function as an oscillator. But for the oscillator to be part of the timekeeping system, it must be able to both receive inputs and generate outputs that convey timing information. In our model, we considered the circadian system and external events as two major time-dependent inputs and derived behavioural actograms from the DA concentration as the output. Our preliminary investigation of the DUSR model suggests that the ultradian DA rhythm integrates mixed signals, organizing daily responsiveness to external stimuli while retaining the ultradian temporal structure.

Our results show a parameter-dependent, biphasic masking effect of the circadian signal on ultradian oscillations. It is important to note that, in our study, the circadian-signal inputs have a one-sided effect on the DUSR rhythm, while biologically the DA system also provides feedback to the circadian system, creating a complex interplay. The transient excitatory input in our model reflects input from the ascending arousal system, conveying subjective perception of important stimuli and potentially guiding goal-directed behaviour. Future research could explore the impact of transient arousing stimulations on coupled oscillators representing the dopamine system and the circadian system. This would provide a more holistic understanding of how these two systems mutually influence each other in different contexts.

For the behavioural output of the DUSR model, the actograms provide a visualization of baseline responsiveness of the motor and motivation DAergic pathways. However, it’s essential to recognize the heterogeneity of the mesostriatal DA system. Excitatory D1 receptors and inhibitory D2 receptors differ in their responsiveness to prolonged DA activation, influencing how the DA signal is interpreted post-synaptically. Different subregions of the striatum and various subnuclei in the midbrain also regulate diverse behaviours, meaning that individual interpretations of DA signal baselines could vary [69].

Midbrain-terminal D2 autoreceptors display a unique lack of internalization upon excess activation in the striatum, suggesting an important role in strictly conveying current DA concentration in the striatum [78]. Our model, balancing structural accuracy with simplicity, offers a generalizable framework for studying ultradian DA rhythms. The model aligns with current knowledge of the double negative feedback loops governing DA release and removal, providing a useful tool for investigating biochemical mechanisms and interpreting behavioural phenomena. Future research includes refining model parameters to emerging experimental data on the presynaptic regulatory processes and applying the model to diurnal animals. Additionally, exploring model reduction through small parameters may help simplify mathematical analysis aimed at determining parameters essential for producing ultradian oscillation and parameters contributing to the oscillation amplitude, which may underlie individual variations in striatal DA measurements [8]. Nevertheless, the rhythmicity responses based on the link between striatal DA oscillations and ultradian behavioural rhythms provide evidence that ultradian rhythms might have had an evolutionary advantage, which we discuss in the following section.

### Functional implications of the simulation results and ultradian rhythms in the timekeeping hierarchy

The pervasiveness and persistence of ultradian oscillations on the physiological level suggest that they are the external manifestation of an internal timekeeping system. It is thus interesting to consider the functional role of ultradian rhythms in the timekeeping hierarchy with their distinct characteristics. The period of the simulated ultradian DA oscillation is easily modified by endogenous parameter variation (Figs 3 and 4), reflecting the large intra- and inter-individual variability reported for ultradian behavioural rhythms [8, 79]. It is also consolidated and confined by the circadian signal, as are the behavioural ultradian rhythms superimposed upon the circadian rhythm. These characteristics of high period flexibility sustained and subject to circadian regulation parallel other locally sustained ultradian rhythms in various biological contexts, such as the pituitary-adrenal regulation and central nucleus mechanisms impacting immune responses, metabolism, and development [4, 45, 80]. These distinct mechanisms share a common structural feature in utilizing negative auto-regulation without additional stabilizing loops commonly seen in circadian clocks. While their specific responses depend on individual properties, they universally orchestrate network dynamics to transition from sustained expression to pronounced ultradian oscillations upon encountering external stimuli. Ultradian rhythms in central mechanisms are suggested to ensure synchronization of individual cellular fate decisions, initiating coordinated responses for group benefit [80]. Ultradian rhythms likely tune to local biological needs alongside the overarching requirements corresponding to the physical light-dark cycle. Dopamine rhythms may involve synchronized responses at the individual organism level for collective benefits. We envision the ultradian behavioural rhythms to facilitate social synchrony among the surrounding biological environment, as living organisms exhibit flexible time-dependent activity patterns within a circadian day.

Synchronized activities offer survival advantages, especially for social animals. Communal animals demonstrate synchronized ultradian rhythms both in laboratory conditions and open fields [81, 82]. Concerted group activities reduce individual risks by providing “safety in numbers”, while synchronized resting aids in heat conservation and resilience in stressful conditions [83, 84]. Synchronized foraging provides increased protection via invigilation and reduces competition for resource distribution [6, 85]. While estrous synchronization tends to be studied on longer time scales, similar reproductive advantages could be generalized to synchronization on the ultradian time scale. In contrast to the intra-species synchronization described above, inter-species social synchrony remains less studied. Nevertheless, it is reasonable to assume that the ability to partially anticipate predator or prey activity would be favoured, given that DA oscillations are involved in fright-driven avoidance and food anticipation [23].

The flexibility of the DUSR provides a foundation for the functional role of ultradian rhythms in social synchrony, prompting us to consider why the ultradian clock is anatomically distinct from the circadian system and built within the DA system. Firstly, it is not uncommon that biological clocks with distinct oscillatory mechanisms co-exist to accommodate diverse requirements and functional roles. Many lower vertebrates, for instance, rely on the dynamic interaction between three photoreceptive clocks that differ in their entrainment responses and output mechanisms to regulate physiological and behavioural variations [86–89]. In mature red blood cells lacking a nucleus to support the transcription-translation feedback loop (TTFL) of clock genes, a persistent oscillation regulating metabolism is maintained via a cytoplasmic redox feedback loop [90, 91]. The distinct clocks differ in their sensitivity and responses to various environmental stimuli as well as in their output mechanisms and targets. The midbrain DAergic neurons, responsible for motor and motivation in spontaneous and goal-directed behaviours, provide a natural platform for the emergence of flexible oscillations. Given their involvement in voluntary interactions, the mammalian DA system is well suited for generating ultradian rhythms, contributing to social synchrony by motivating engagement in recurring events.

Social events recur within a day and the ultradian rhythm is inevitably contextualized by circadian entrainment. The DA system likely relies on the circadian clock to establish stable rhythms that could be utilized by the host, as indicated by the masking and coupling effect of a circadian signal on the DUSR rhythm (Figs 5 and 6). Indeed, daily light onset has a strong synchronizing effect on ultradian behaviour [83]. Clock-controlled genes, with their rhythmic concentration, exert time-varying influences on DA self-regulation. Our model incorporates circadian influence at the oscillator level, but its impact extends to both the input and output of DUSR. Social synchrony can either arise directly through entrainment among individuals or indirectly through individual entrainment to a shared circadian environment. In the former case, coordinated behaviours constitute both the input and output of the ultradian oscillator, while in the latter case, it is solely the output. It would be more functionally efficient if the ultradian oscillator coordinating social synchrony is directly responsive to significant interactions among the surrounding biological environment. But as all functional advantages are adapted to the context of the surrounding physical environment, the ultradian oscillations should also adapt to the changing requirements depending on the circadian time of day. We propose that the DUSR acts as a coordinator between the biological and physical environment, receiving inputs of circadian regulation and emotional arousal experiences.

The distinct responses of DUSR to both inputs do not result from a simple additive effect, but rather reflect a coordinated response to their interactive effect (Fig 8). Within the ultradian oscillatory range, while the circadian coupling suppresses pre-existing ultradian oscillation, it increases the DUSR’s response to a transient stimulus, reflecting increased sensitivity to significant experiences. It also reorganizes responses to stimuli, consolidating them during the individual’s active phase of the day. This extended response to single significant experiences helps maintain synchrony when the input is not constant, such as in social synchrony between non-cohabiting individuals. We predict from our results that the ultradian rhythm is the default rhythm of the DA system, but it requires circadian regulation to fulfill its functional role. Animals with acquired circadian-arrhythmicity might have a pronounced ultradian rhythm but a reduced ability to maintain socially synchronized behaviours, leading to reduced entrainment to social interactions and an increased tendency to desynchronize from companions. Further animal experiments can confirm or refute these predictions and clarify the role of ultradian rhythms in the timekeeping hierarchy.

## Model and methods

### Core model equation description

The core DUSR model consists of Eqs (1)–(4) and focuses on the DA-D2 self-regulatory processes that form the core ultradian oscillator. A summary of the time-dependent external inputs to the DUSR and a possible way to translate DA concentration to behavioural output will be described in the sections following the analysis of the core model. A Matlab .m and an XPPAUT .ode of the model is provided in the Supporting Information S1 Files.

#### Extracellular DA concentration dynamics

Extracellular DA in the striatum is primarily regulated by two processes: release through vesicular exocytosis initiated by successful action potentials and removed through dopamine transporter (DAT)-dependent reuptake [50, 92]. Both processes bias DA concentration between the cytosolic and the extracellular space across the pre-synaptic membrane.

For DA release, we assume a sufficient pre-synaptic vesicular DA storage and approximate the change rate in DA concentration *DA*^ex^ as linearly dependent on two factors: the state-derived variable *F*, which represents neuron firing frequency, and the rate constant *α*, which represents the unit increase in *DA*^ex^ elicited by a single population firing event. A range of values for *α*, 117 ± 11/13 nm in the caudate putamen and 78/80 ± 8/7 nm in the nucleus accumbens respectively, have been measured in [48]. As our model focuses on general DA behaviour in the entire striatum, we take around the average value *α* = 0.09 μm.

For DA removal, the uptake rate of *DA*^ex^ is given by the Michaelis-Menten kinetics ($\frac{V_{max}\cdot [S]}{K_{M}+[S]}$) with *DA*^ex^ as the substrate concentration [S]. For consistency, the DA uptake and DA release parameter values are extracted from the same studies [48, 49], such that *K*_M_ = 0.2 μm and *V*_max_ = 4 − 5.5 μm s^−1^ in the caudate and 2.5 − 3.5 μm s^−1^ in the nucleus accumbens. *K*_M_ reflects the affinity of DA to DAT and exhibits minimal spatiotemporal variation, while *V*_max_ reflects the maximum achievable rate determined by transporter availability and is susceptible to D2 autoreceptor activation [93]. *V*_max_ is thus made to depend on the state variable *T*_DA_ such that *V*_max_ = *k*_Vmax_ ⋅ *T*_DA_. The value range for *k*_Vmax_ is derived from dividing the *V*_max_ values by *T*_DA_ = 1.4, corresponding to when DAT is at the midway of its availability range. The parameter value *k*_Vmax_ = 2.63 × 3600 μm h^−1^ is selected through simulation and gives a *V*_max_ value that oscillated between 2.75 and 3.55 μm s^−1^.

Compared to the exocytotic release and DAT-dependent reuptake, synaptic and post-synaptic processes are slow and contribute insignificantly to striatal DA kinetics. For the DUSR model, we simplify their combined rate into a first-order kinetics proportional to *DA*^ex^ with a rate coefficient *β* = 144 h^−1^ based on the reported DA clearance rate of 0.02 − 0.04 s^−1^ in DAT knock-out mice [50, 51]. Being only approximately 0.3% of the reuptake rate (linearized as $\frac{k_{Vmax}}{K_{M}}$), *β* has minimal impact on all results, as expected for a parameter not involved in the negative feedback loops.

#### D2 autoreceptor activation

Eq (1) captures the binding-dissociation kinetics of DA and the D2 autoreceptors. Upon binding with DA, D2 switches from the free, inactive state to the fully activated state in the form of a ligand-receptor complex. This process is reversible,

$$\underset{\text{inactive}\;\text{autoreceptors}}{D2}+DA\overset{\text{binding}}{\underset{\text{unbinding}}{⇌}}\underset{\text{activated}\;\text{autoreceptors}}{D2-DA}.$$

As the active state of D2 depends on the occupancy of a single binding site, a first-order reaction determines the equation. The rate at which D2 becomes occupied by DA is dependent on the ligand concentration *DA*^ex^, the concentration of free D2 receptors (*D*2^tot^ − *D*2_AR_), and the rate constant *k*. *D*2^tot^ represents the total concentration of membrane-bound D2 autoreceptors at the striatum and *D*2_AR_ represents the concentration of occupied D2 autoreceptors. The rate *k* reflects both the diffusion rate of DA to autoreceptor sites and the binding coefficient of DA to D2. The unbinding rate of D2-DA complexes is assumed proportional to its own concentration *D*2_AR_ and governed by the dissociation coefficient *a*.

A previous model of striatal D2-binding [47] estimated striatal D2 concentration (*D*2_tot_) as approximately 0.1 μm with a DA dissociation constant (*K*_d_) of 25 nm^−1^. However, the reliability and reproducibility of biochemical measurements have been longstanding challenges [94, 95], with reported D2-DA binding kinetics varying up to hundredfold between experiments (see in: PDSP/IUPHAR database). Moreover, many experiments did not differentiate between D2’s subcellular location against the synapse. Given the substantial variability in reported D2 kinetics and the exclusive focus of our model on pre-synaptic D2 autoreceptors, we primarily determined our kinetic parameter values based on simulation. As D2 activation paves the foundation step for both DA self-feedback loops, concurrently adjusting both constants with the same factor modifies the oscillation period. The parameter values *D*2_tot_ = 0.1 μm, *k* = 10.46 μm^−1^ h^−1^, and *a* = 1.7 h^−1^ result in an average D2 occupancy of around 20% when *DA*^ex^ = 0.04 nm, consistent with literature estimates of baseline striatal D2 autoreceptor occupancy in the lower tenth [30, 62, 63].

#### Pre-synaptic electrical activity

To capture the dynamics of impulse-dependent DA release, midbrain DAergic neuron activity is modelled on the population level and represented by *V*_0_, the average resting membrane potential. Accounting for all the excitatory and inhibitory inputs to midbrain DAergic neurons is unrealistic and will unnecessarily complicate the model. As our model emphasizes the evolution of DA neurodynamics over time, the time-dependent inputs are extracted and the summarized effect of other inputs is represented as a baseline firing rate corresponding to the baseline membrane potential at *V*_0_ = 0 mV. Eq (2) describes the evolution of *V*_0_ over time due to self-regulation [52], which we describe on in this section. The effects of time-dependent inputs from other brain regions onto midbrain DAergic neurons will be discussed in later sections.

Neurons have a tendency to restore their electrochemical equilibrium maintained through K^+^ disequilibrium within a certain time scale. This restoring rate is proportional to *V*_0_ at a rate constant *c*, which is the reciprocal of the decay time. In the absence of further stimulations, *V*_0_ approaches its baseline exponentially. D2 autoreceptors exert a concentration-dependent inhibitory control on pre-synaptic neuron excitability by increasing membrane potassium conductance [39, 96] and decreasing membrane calcium conductance [41, 97]. We summarize this D2-dependent inhibitory effect on *V*_0_ to be a rate constant *k*_V_ times the current *D*2_AR_ value. In addition to the D2-dependent inhibitory regulation through negative feedback, midbrain DAergic neurons also exhibit D2-independent excitatory coupling through positive feedback [98–100]. These excitatory effects are defined as proportional to the current firing rate *F* times a rate constant *b* that reflects the conversion strength from neuron activity to neuron electrochemical state. The value of *F* is determined instantaneously from *V*_0_ as described in the following paragraph. Parameter values *c* = 3.62 h^−1^, *b* = 0.012 mV, *k*_V_ = 2.73 × 3600 mV μm^−1^ h^−1^ are manually selected through simulation to reproduce an ultradian period of 4.0 h.

The firing rate of midbrain DAergic neurons alters between a low-rate tonic firing of below 4 Hz and a high-rate burst-firing capped at 15 Hz [46, 53, 56, 101–103]. The mean neuron firing rate *F* represents the population firing behaviour and is approximated as a sigmoid function of *V*_0_, whose midpoint and slope steepness are determined by parameters *θ* and *σ* respectively. *θ* represents the input *V*_0_ value at which the output *F* is halfway towards the maximum and reflects the averaged population firing threshold relative to resting. *σ* determines how quickly *F* saturates towards the maximum and reflects the threshold variation among individual neurons. Past experiments suggest that an action potential could be triggered in DAergic neurons when the membrane potential depolarizes 15 to 30 mV above resting potential and reported a lower variation among individual midbrain DAergic neurons [54–56]. With the chosen parameter values *F*_max_ = 15 Hz, *θ* = 25 mV, and *σ* = 18 mV, *F* has the value of 2.98 Hz when *V*_0_ is at its baseline value, such that *F* remains well below the tonic firing rate when *V*_0_ is below the baseline and then saturates exponentially fast to *F*_max_ with increasing *V*_0_.

#### Dopamine transporter activity

Eq (3) describes the kinetics of the dimensionless variable *T*_DA_, the availability of dopamine transporters (DAT) expressed as the ratio against its minimal availability in the striatum. DA transporters are constantly translocated between the internal cytoplasm and the surface membrane of pre-synaptic terminals, where they actively participate in removing DA from the extracellular space [104, 105]. D2 activation contributes significantly to the short-term modification of DAT cell-surface expression by accelerating its forward-trafficking to the membrane (see review: [106]), such that increasing *D*2_AR_ by increased *DA*^ex^ leads to increased *T*_DA_ and thus increased removal of DA from the extracellular space [35, 93]. We approximate *T*_DA_ from *D*2_AR_ with a sigmoidal curve between its minimum (*T*_*DA*_ = 1) and maximum availability (*T*_DA_ = Δ*T*). The additional availability to the minimum contributed by *D*2_AR_ is $\frac{\Delta T-1}{1+e^{-k_{T}(D2_{AR}-D_{0})}}$, which is a positive value bounded above by Δ*T* − 1. The most rapid increase in *T*_DA_ occurs at *D*2_AR_ = *D*_0_, which reflects the half-efficiency of D2 autoreceptors on transporter regulation. The steepness of the slope is determined by the parameter *k*_T_ and, additionally, *T*_DA_ requires a time delay of *τ*_T_ to reach the new dynamic equilibrium.

Exciting the D2 regulatory pathway can increase cell-surface DAT expression to over 150% and inhibiting the pathway decreased cell-surface DAT by almost 50% [34, 35]. These changes in surface DAT ratio peaked between 5 to 15 min after the evoked D2 regulation and lasted for less than an hour. Based on these findings, parameter values Δ*T* = 1.8 and *τ*_T_ = 0.15 h^−1^ are chosen for the current model. As D2 agonists induced a larger relative change in membrane-bound DAT ratio from baseline than did D2 antagonists, *D*_0_ = 0.04 μm and *k*_T_ = 87.5 μm^−1^ were chosen so that the simulated *D*2_AR_ values remain below *D*_0_.

### Simulation, local sensitivity, and bifurcation analysis

Model simulations were performed with an ODE solver in Matlab 9.9.0 (The Mathworks, Inc. Natick MA). To investigate the rhythmic behaviour of the model under different endogenous conditions, local sensitivity analysis is performed on all parameters at a nominal period of 4.0 hours using the equation,

$$\begin{array}{c} \text{Period}\;\text{Sensitivity}\;\text{Coefficient}=\frac{d(\text{Period})}{d(\text{Param})}\frac{\text{Nominal}\;\text{Param}}{\text{Nominal}\;\text{Period}}. \end{array}$$ (7)

The period sensitivity is estimated under a perturbation of 1% for each parameter.

To further characterize the stability of the ultradian oscillation and determine the range of ultradian period that could be generated by the model upon modification of individual processes, bifurcation analysis was performed with XPPAUT AUTO [107] (Ntst = 60; Ds = 0.01; Dsmin = 0.005; Dsmax = 0.05; other parameters at the default value). Simulations started at a stable equilibrium below and above the oscillatory range containing the chosen value. The results were verified with Matlab simulations at various values chosen along the continuation curve.

### Effects of circadian inhibitory inputs on DA oscillations

The solar day is inarguably the most important rhythm for terrestrial mammals and multiple modulatory signals on the DA system display a circadian rhythm (see review: [66]). The master circadian clock located in the SCN solely determines the presence and the period of circadian rhythms in spontaneous behavioural activity, entraining endogenous rhythms and peripheral clocks located throughout the brain and body [108]; core clock genes expressed within midbrain DAergic neurons alter phenotype at the behaviour level [109, 110]; the midbrain-striatum circuitry comprising mesostrial DAergic projections and retrograde striatomesencephalic pathways plays a crucial role in the food-entrained and methamphetamine-induced circadian rhythms [1]. In addition to circadian modulation on behaviour, mathematical simulations in [44] further showed that the circadian molecular clock regulating DA synthesis and degradation leads to circadian rhythmicity in extracellular DA concentration. Because the overt circadian rhythms of healthy individuals are normally synchronized, a single time-dependent term *R*_circ_(*t*) is used to represent the collective circadian regulatory strength acting upon the DUSR. The value *R*_circ_ oscillates between 0 and the maximum at an endogenous period of *T*_circ_ = 24 h.

In nocturnal rodents, SCN neural activity has an antiphase relationship with behavioural activity and suppressing SCN activity induced behavioural activity [65]. Physiologically, connections from the SCN and the striatum to the midbrain are mostly inhibitory [66, 111]. We thus append the circadian regulatory signal as a negative term to Eq (2), so that the ODE for *V*_0_ becomes

$$\begin{array}{c} \frac{dV_{0}}{dt}=-cV_{0}+b\cdot F-k_{V}D2-R_{\text{circ}}\left(t\right). \end{array}$$ (8)

The modified system of ODEs is simulated with *R*_circ_(*t*) as either a 12 h:12 h switch function or a sine function reflecting the sinusoidal waveform of SCN neural activity [65, 67]. As SCN neural activity may exhibit asymmetry between its daily rise and fall phases, we sought to explore the effect of the circadian signal’s waveform on ultradian rhythm regulation by simulating *R*_circ_(*t*) using both standard and tilted sine functions.

### Effects of transient excitatory inputs on DA oscillations

Mesostriatal DA contributes to the execution of cue-triggered and goal-directed behaviours. Encountering a single emotionally arousing event can have a time-dependent effect on DA-associated behaviour [112]. Midbrain DAergic neurons projecting to the striatum receive selective excitatory and dis-inhibitory inputs from cholinergic neurons, which encode perceived saliency and participate in motivated behaviour [69, 113–115]. These inputs increase striatal extracellular DA by concentration increasing impulse-triggered DA release [69, 70, 115, 116]. We modelled a transient salient stimulus as a single rectangular pulse *I*_sens_ spanning the duration of an emotionally arousing event. This stimulus acts as an excitatory input that increases the average membrane potential of the DAergic neurons as

$$\begin{array}{c} \frac{dV_{0}}{dt}=-cV_{0}+b\cdot F-k_{V}D2+I_{\text{sens}}\left(t\right). \end{array}$$ (9)

Circadian rhythms and salient stimulus interact at striatal DA to facilitate timestamping and anticipating the recurrence of important events, hypothesized to involve an increase in behavioural motivation [23, 112]. To gain insight into the combined effect of both a circadian-rhythmic inhibitory signal and a transient excitatory signal, the DUSR is simulated with both *R*_circ_ and *I*_sens_ appended, with the circadian time when *I*_sens_ occurs chosen to depend on the phase of *R*_circ_:

$$\begin{array}{c} \frac{dV_{0}}{dt}=-cV_{0}+b\cdot F-k_{V}D2-R_{\text{circ}}\left(t\right)+I_{\text{sens}}\left(t\right). \end{array}$$ (10)

To ensure complete processing of the brief stimulus, integration was stopped and restarted at the times of the discontinuous input within the ODE solver simulation.

### DA oscillations and behavioural activity

Behavioural rhythms are hypothesized to be both a physiological indicator and a regulated output of striatal DA rhythms. We thus sought to reproduce behavioural activity patterns from the simulated dynamics in DA concentration. However, the relationship between activity level and DA concentration is mostly established in phase rather than in amplitude. Episodes of behavioural activity are correlated with and preceded by a local peak in the striatal DA concentration, rather than when DA exceeds a fixed threshold [8]. Striatal DA concentration displays considerable intra-individual variation and rises drastically upon administration of psychostimulants, while the changes in behavioural activity level are comparatively modest. This could be due to a combined limiting and desensitizing effect of both post-synaptic D1 and D2 receptors, as well as downstream pathways. It is thus difficult to predict whether an animal is active or how active an animal will become from an isolated measurement of DA.

To convert the simulated striatal extracellular DA concentration values to behavioural activity while accounting for the lack of a quantitative relationship, DA has to serve as its own control. We applied a circadian moving threshold that outputs only two states, active (1) and inactive (0), to the simulated DA values. The current *DA*^ex^ value is compared to the time-averages *DA*^ex^ value over the previous circadian cycle (Δ*T* = *T*_circ_ = 24 h) and the output is regarded as active if it surpasses the average. Therefore, we define activity as

$$\begin{array}{c} \text{Activity}\left(t\right)=\{\begin{array}{cc} 0, & DA^{\text{ex}}\left(t\right)\leq {\left\langle DA^{\text{ex}}\left(t\right)\right\rangle }_{\Delta t}\\ 1, & DA^{\text{ex}}\left(t\right)>{\left\langle DA^{\text{ex}}\left(t\right)\right\rangle }_{\Delta t} \end{array}. \end{array}$$ (11)

Behavioural activity is visualized as double-plotted actograms starting from *t* = 24 h with the Actiview software (version 1.2, Mini Mitter Co., Inc., Oregon, USA).

## Supporting information

S1 Files Model code. The model implemented in Matlab file DUSR.m and XPPAUT file DUSR.ode.
(ZIP)
